# Beyond Borders: Exploring the Quality of Life, Health-Seeking Behavior, and Perceived Barriers in Health Services Utilization Among Sudanese Immigrants in Egypt

**DOI:** 10.7759/cureus.52442

**Published:** 2024-01-17

**Authors:** Sameer H Hamdy

**Affiliations:** 1 Community Health Nursing, Beni-Suef University, Faculty of Nursing, Beni-Suef, EGY; 2 Community and Mental Health Nursing, Najran University, Faculty of Nursing, Najran, SAU

**Keywords:** health-seeking behavior, health services utilization, egypt, quality of life, immigrant sudanese

## Abstract

Background: The detention of immigrants has adverse effects on both their health and their overall quality of life. In migration conditions, there is a notable impact on health-seeking behavior.

Objective: This study aims to explore the quality of life, health-seeking behavior, and perceived barriers to health services utilization among Sudanese immigrants in Egypt.

Materials and methods: The study employed a cross-sectional design to fulfill its objectives. A convenience sample comprising 385 Sudanese immigrants post-war was utilized. The researcher employed a structured questionnaire consisting of four parts: socio-demographic data, a quality of life questionnaire, an assessment of health-seeking behavior, and an exploration of the barriers faced by Sudanese immigrant participants in accessing health services.

Results: The study on Sudanese immigrants post-war revealed diverse perceptions of overall quality of life, with 41.6% reporting satisfaction, 32.4% an average state, and 26.0% unsatisfactory conditions. Factors such as gender, educational level, financial situation, and the primary reason for relocating to Egypt significantly influenced these outcomes (p<0.05). Encouragingly, 57.1% of the immigrants exhibited positive health-seeking behavior. Noteworthy barriers to healthcare access included limited awareness of available services (60.5%), competing priorities like work (53.2%), insufficient financial resources (49.6%), and extended waiting times in healthcare facilities (45.5%). These findings underscore the complex interplay of factors affecting the well-being and health-seeking patterns of Sudanese immigrants in their new environment.

Conclusion: The research sheds light on key aspects of the well-being of Sudanese immigrants in Egypt, offering insights into their quality of life, health-seeking behavior, and perceived barriers to healthcare. The findings reveal a diverse spectrum of overall quality of life, with over a third expressing satisfaction, while a significant portion reported average or unsatisfactory levels. The study underscores the intricate influence of gender, educational level, financial situation, and the primary reason for immigration on the quality of life. Positive health-seeking behavior was observed in more than half of the participants, yet the identification of barriers to healthcare access signals challenges that warrant attention for enhancing overall health outcomes among this immigrant population. Implementing community-based health education programs plays a pivotal role in empowering immigrant Sudanese individuals in Egypt to make informed health choices and adopt healthy lifestyles.

## Introduction

Migration involves the movement of people within a country or across international borders, encompassing various forms regardless of duration, composition, or purpose [1]. The process of social and demographic change is pivotal, with international migration reaching unprecedented levels in recent decades. In 2019, the International Organization for Migration estimated that 272 million people were international migrants, residing outside their birthplaces. While globalization has facilitated migration, making it more accessible and affordable, people are still compelled to leave their homes due to factors such as violence, poverty, and the impacts of climate change [2].

Migrants, especially those lacking proficiency in the host language, encounter various obstacles to maintaining good health in their new country. This limitation is identified as a significant factor contributing to low levels of health-seeking behavior, ultimately resulting in suboptimal health outcomes. Health-seeking behavior is broadly defined as actions taken to care for and preserve one's health, irrespective of current health status. This encompasses health-promoting practices like making healthy food choices, engaging in regular physical activity, participating in health promotion and education programs, and accessing preventive health services such as cancer screening [3].

Migration involves individuals encountering diverse cultures, lifestyles, and healthcare systems as they settle in new areas. These interactions, inherent in adapting to new environments, may create situations with potential negative impacts on health. Understanding the dynamics of health-seeking behavior in migrant communities is gaining significance. Recent research underscores the importance of exploring cultural influences, socio-economic factors, and structural barriers that shape migrants' health decisions [4]. This recognition emphasizes the complexity of health dynamics within migrant populations and the need for a nuanced understanding to address their unique healthcare challenges.

Quality of life, extending beyond conventional measures, now embraces a holistic perspective that includes social integration, mental health, and physical well-being. Recent research underscores the importance of recognizing the distinct barriers to quality of life experienced by immigrant communities, emphasizing the necessity for tailored interventions that address their individual needs and goals [5]. Comprehensive studies indicate that the health-related quality of life (HrQoL) for migrants often tends to be lower compared to native-born individuals [6]. Immigrants frequently navigate a challenging balance between adapting to a new cultural environment and overcoming obstacles tied to their migration experiences [7].

The Sudanese conflict has triggered mixed cross-border movements, with 926,841 people seeking refuge in neighboring countries. Of this, over 283,500 individuals have fled to Egypt for protection, as reported by the Egyptian Ministry of Foreign Affairs as of August 1, 2023 [8]. The socio-political and cultural milieu of Egypt significantly influences the experiences of Sudanese migrants. Recent studies emphasize the pivotal role of host countries in shaping immigrant health outcomes, emphasizing the need to comprehend the local context for developing effective support systems [9].

The current study aims to investigate the quality of life, health-seeking behavior, and perceived barriers to healthcare services utilization among Sudanese immigrants in Egypt. The current study has three research questions: What are the quality of life levels experienced among Sudanese immigrants residing in Egypt? How do Sudanese immigrants in Egypt navigate and engage in health-seeking behaviors within the local healthcare system? What are the primary barriers and challenges that Sudanese immigrants face when utilizing health services in Egypt?

## Materials and methods

Study design and setting, sampling technique, and sample size

The research employed a cross-sectional study design and was conducted in Cairo, the capital of Egypt. A convenience sample was used. The study meticulously determined a sample size of 385 participants through a systematic calculation utilizing the well-established sample size estimation formula. By applying the formula: sample size = (1.962) x (50) x (1-0.5) / 0.052, the researchers took into account critical parameters including the desired confidence level (1.96 for a 95% confidence interval), the margin of error (0.052), and the estimated proportion of the population with a particular characteristic, in this instance, set at 50%.

Inclusion and exclusion criteria

The inclusion criteria for this study encompass individuals aged 18 years and older who are presently residing in Cairo and have migrated due to war. Additionally, participants must have willingly agreed to take part in the study to be considered eligible. Conversely, the exclusion criteria entail individuals who do not meet the aforementioned inclusion criteria, thus emphasizing the importance of fulfilling these conditions for participation.

Data collection instruments

The data collection tools for this study were meticulously designed to gather comprehensive information, with a focus on the unique circumstances of Sudanese immigrants in Egypt. A structured questionnaire, developed by the researcher following an exhaustive review of pertinent literature, constituted the primary instrument. This questionnaire featured four distinct parts, each serving a specific purpose. The first part was dedicated to obtaining socio-demographic information, delving into essential details such as age, gender, educational level, occupation, income, and length of stay. The second part focused on assessing the quality of life among Sudanese refugees, comprising 23 questions that covered various aspects, including social conditions, physical health, psychological well-being, housing conditions, and lifestyle. The questionnaire drew upon existing literature for question inspiration, incorporating insights from studies by [6,10,11]. A scoring system was employed, categorizing responses into scores of "1" for positive answers and "0" for unhealthy responses. The third part, centered on health-seeking behavior, incorporated nine questions derived from studies by [3,12,13]. The fourth part collected data on barriers to healthcare and suggested facilitators, utilizing close-ended questions with predefined options and allowing participants to add additional responses.

The scoring system for the quality of life section, with a total score of 16, enabled the conversion of results into percentages and subsequent categorization into unsatisfactory (<50%), average (50-74%), and satisfactory (≥75%) levels. Similarly, the health-seeking behavior section utilized a scoring system with a total score of 9, categorizing participants' answers as positive health-seeking behavior if the score was above 50%. The inclusion of these well-defined categories enhances the precision and interpretability of the collected data.

Moreover, the fourth part regarding barriers and suggested facilitators utilized close-ended questions with predefined options. Participants were also given the flexibility to contribute additional responses not listed in the options, allowing for a more nuanced understanding of the challenges and potential solutions perceived by the Sudanese immigrant community in Egypt. This thoughtful approach to data collection ensures a holistic exploration of the health-related experiences and perceptions within the study population.

Validity and reliability

The validation and reliability of the questionnaire employed in this study were subjected to rigorous evaluation, underscoring the commitment to ensuring the robustness of the data collection instruments. To achieve this, a panel of five professors with expertise in community health nursing and psychiatric nursing departments undertook the validation process, contributing diverse perspectives and specialized knowledge. The validation process involved a meticulous examination of the questionnaire's content, structure, and relevance to the study objectives. The panel's collective expertise ensured a comprehensive evaluation that encompassed various dimensions of the instrument.

To assess the internal consistency of the questionnaire, particularly in the quality of life and health-seeking behavior sections, Cronbach's alpha coefficient test was employed. The calculated coefficient for the quality of life section yielded a high level of internal consistency, with a Cronbach's alpha coefficient of 0.91. Similarly, the health-seeking behavior questionnaire demonstrated robust internal consistency, with a Cronbach's alpha coefficient of 0.86. These coefficients indicate a strong correlation among the items within each section, affirming the reliability of the questionnaire in consistently measuring the intended constructs. The high Cronbach's alpha coefficients reflect the internal reliability of the questionnaire, instilling confidence in the precision and accuracy of the collected data. Overall, the thorough validation process and the demonstration of strong internal consistency contribute to the credibility and trustworthiness of the questionnaire as a reliable tool for assessing the quality of life and health-seeking behavior among Sudanese immigrants in Egypt.

Data collection procedures

The electronic questionnaires were distributed using a multi-faceted approach. We employed social media platforms and online community forums to reach potential participants. The electronic questionnaire distribution spanned from May 2023 to September 2023, offering a substantial timeframe designed to capture a comprehensive snapshot of Sudanese immigrants' quality of life, health-seeking behavior, and encountered barriers in health services utilization. This extended duration was strategically chosen to allow for a thorough and representative exploration of the study's objectives. The timeline ensured that the survey covered a broad spectrum of experiences and perspectives within the specified timeframe, contributing to the richness and depth of the collected data. This deliberate approach aimed to accommodate potential variations in participants' responses, considering factors such as seasonal influences, individual circumstances, and potential changes in health-related behaviors over time. By adopting this extended timeframe, the researchers aimed to enhance the validity and reliability of the study outcomes, providing a nuanced understanding of the complex dynamics surrounding the health and well-being of Sudanese immigrants in Egypt during the specified period.

Ethical considerations

The ethical considerations guiding this research were meticulously adhered to, with a paramount focus on safeguarding the welfare and rights of the participants. The Research Ethical Committee, Faculty of Medicine, Beni-Suef University has approved the protocol from the ethical point of view (approval number: FMBSUREC/12062023). Prior to data collection, participants were provided with a comprehensive explanation of the study's aims, procedures, and potential implications. Written informed consent was then obtained from each participant, signifying their voluntary agreement to participate in the research. This transparent and informed consent process was crucial in upholding the principles of autonomy and respect for participants' decisions.

Statistical analysis

SPSS Statistics version 24.0 (IBM Corp. Released 2016. IBM SPSS Statistics for Windows, Version 24.0. Armonk, NY: IBM Corp.) served as the analytical tool, facilitating the extraction of meaningful information from the amassed data. To discern significant differences between the variables investigated, the Chi-square test and Spearman's correlation test were used. This method allowed for the identification of patterns and associations within the dataset, contributing to a more nuanced understanding of the relationships between different factors.

## Results

The frequency distribution in Table 1 provides a snapshot of the socio-demographic characteristics of the studied sample. The age distribution revealed a relatively balanced representation, with <25 years (28.6%), 25-40 years (42.6%), and >40 years (28.8%). There was a gender imbalance, with males accounting for 58.4% and females for 41.6%. Educational levels showed diversity, comprising primary education (20.8%), secondary education (32.5%), and university education (46.8%). Respondents' self-reported financial situations are represented by percentages as follows: good (54.5%), fair (24.7%), and poor (20.8%). The distribution of respondents with and without relatives in Egypt before the war is represented by percentages: yes (49.1%) and no (50.9%). In relation to the main reasons for coming to Egypt, percentages were presented: 57.1% for permanent residence until the war ends, and 42.9% for a transitional period until traveling to another country.

**Table 1:** Frequency distribution of the studied migrants based on socio-demographic data

Items	N	%
Age		
<25	110	28.6
25-40	164	42.6
>40	111	28.8
Gender		
Male	225	58.4
Female	160	41.6
Educational level		
Primary	80	20.8
Secondary	125	32.5
University education	180	46.8
How would you describe your current financial situation?		
Good	210	54.5
Fair	95	24.7
Poor	80	20.8
Do you have relatives who lived in Egypt before the war?		
Yes	189	49.1
No	196	50.9
What is the main reason to come to Egypt?		
Permanent residence until the war ends	220	57.1
A transitional period until I can travel to another country	165	42.9

As shown in Table 2, the majority of respondents (47.5%) reported a satisfactory quality of life in the physical domain, while 30.4% rated it as average and 22.1% found it unsatisfactory. Regarding the psychological domain, a significant portion (38.4%) perceived their psychological well-being as satisfactory, with 26.5% rating it as average and 35.1% finding it unsatisfactory. The social domain shows a positive perception, with 51.9% reporting satisfactory quality of life, 33.8% as average, and 14.3% as unsatisfactory. In relation to housing conditions, housing conditions yielded varied responses, with 31.2% reporting satisfaction, 29.9% rating it as average, and 39.0% finding it unsatisfactory. The lifestyle domain indicates diverse perceptions, with 28.6% reporting a satisfactory quality of life, 36.4% as average, and 35.1% as unsatisfactory. Finally, the overall quality of life is summarized, with 41.6% reporting satisfactory, 32.4% average, and 26.0% unsatisfactory.

**Table 2:** Quality of life of studied immigrant Sudanese

Domains	Satisfactory		Average		Unsatisfactory	
	N	%	N	%	N	%
Physical	183	47.5	117	30.4	85	22.1
Psychological	148	38.4	102	26.5	135	35.1
Social	200	51.9	130	33.8	55	14.3
Housing condition	120	31.2	115	29.9	150	39.0
Lifestyle	110	28.6	140	36.4	135	35.1
Total quality of life	160	41.6	125	32.4	100	26.0

The results presented in Table 3 provide an overview of health-seeking behaviors among the immigrant Sudanese population. The table outlined different actions participants would take when facing health problems. Only about one-fifth visited health services, a significant proportion (32.5%) resorted to self-medication, and 28.6% selected alternative medicines. About 16.9% of respondents indicated a tendency to delay addressing health problems. About half of the respondents engaged in periodic health screenings, with high percentages for measuring blood pressure (49.4%) and blood sugar (48.6%). A relatively low percentage (11.7%) reported monitoring the growth and development of their children. A substantial number of respondents (36.4%) reported vaccinating eligible family members. About 23.4% reported following antenatal care for themselves or their family members. The table summarizes health-seeking behavior into positive (42.9%) and negative (57.1%) categories.

**Table 3:** Health-seeking behavior among the immigrant Sudanese

Items	N (yes)	%
First action that will be taken by the participants when getting any health problems		
Visit health services	85	22.1
Self-medication	125	32.5
Alternative medicine	110	28.6
Delay the issue	65	16.9
Screening for general health		
Measuring blood pressure periodically	190	49.4
Measuring blood sugar periodically	187	48.6
Dental checkup	65	16.9
Visual checkup	40	10.4
Cholesterol screening	38	9.9
Use maternal and child health services		
Did you monitor the growth and development of your children?	45	11.7
Did you vaccinate children and any eligible family members?	140	36.4
Did you or a member of your family follow antenatal care?	90	23.4
Total levels		
Positive health-seeking behavior	165	42.9
Negative health-seeking behavior	220	57.1

Table 4 shows that a substantial portion of respondents (45.5%) identified long waiting hours in healthcare facilities as a barrier to seeking healthcare. Almost half of the respondents (49.6%) cited a lack of financial resources as a significant barrier to seeking healthcare. More than half of respondents (53.2%) expressed that competing priorities, like work, pose a barrier to seeking healthcare. A significant number of respondents (60.5%) highlight limited awareness of available services as a barrier to seeking healthcare. A notable percentage of respondents (19.7%) identify stigma and discrimination as barriers to healthcare seeking.

**Table 4:** Frequency distribution of the studied migrant regarding perceived barriers to health-seeking behavior

Items	N	%
Long waiting hours in a healthcare facility	175	45.5
Lack of financial resources	191	49.6
Competing priorities like work	205	53.2
Limited awareness of available services	233	60.5
Stigma and discrimination	76	19.7

Table 5 reveals that about two-thirds (64.2%) of Sudanese immigrants express a need for culturally competent healthcare providers. The majority of immigrants (72.2%) advocate for inclusive healthcare policies. A substantial number of immigrants (84.9%) emphasize the importance of flexible clinic hours. A notable but comparatively lower percentage (18.2%) of Sudanese immigrants mentioned legal protections for non-discrimination as a suggestion to improve health-seeking behavior. The majority of Sudanese immigrants (81.8%) stress the importance of announcements about available healthcare services.

**Table 5:** Frequency distribution of the studied migrant regarding your suggestions to improve health-seeking behavior

Items	N	%
Culturally competent healthcare providers	247	64.2
Inclusive healthcare policies	278	72.2
Flexible clinic hours	327	84.9
Legal protections for nondiscrimination	70	18.2
Announce the available healthcare services	315	81.8

**Figure 1:**
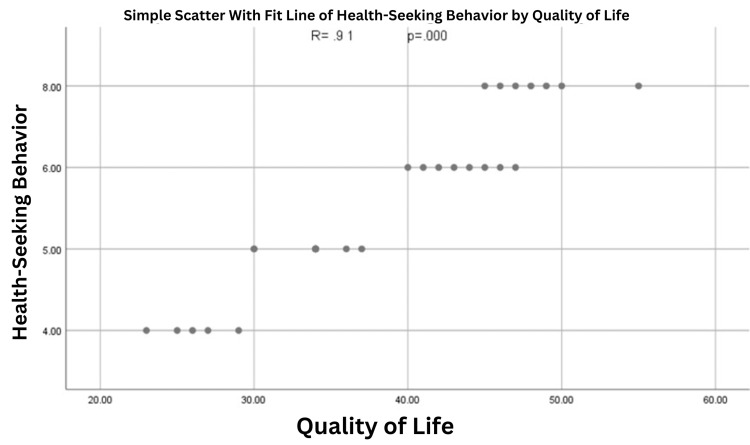
Correlation between the health-seeking behavior of Sudanese immigrants' and their quality of life

Figure 1 illustrates the correlation between the health-seeking behavior of Sudanese immigrants' and their quality of life. The Pearson correlation showed that R=0.91 and p=0.000, indicating a positive and significant correlation between health-seeking behavior and quality of life among the immigrant Sudanese.

As shown in Table 6, the Chi-square test results (X2=5.1, p=0.27) indicate that there is no significant association between age and quality of life levels. There is a clear gender imbalance, with a higher number of males (225) compared to females (160) in the sample. The chi-square test (X2=20.9, p=0.0003) reveals a significant association between gender and quality of life levels. Our findings revealed that male immigrants were more likely to report satisfactory QOL compared to their female counterparts. This association may be attributed to various factors, including societal expectations, cultural roles, and access to resources. The chi-square test (X2=54.5, p=0.00001) indicates a significant association between educational level and quality of life. Additionally, the table indicates a strong association (X2=66.0, p=0.00001) between the reported financial situation and quality of life levels. This association could be due to employment opportunities, the cost of living, and access to resources being better among highly educated people and those who had high incomes. Immigrant Sudanese who came for permanent residence in Egypt were observed to have significantly higher levels of quality of life than those who came for a transitional period (p=0.00001).

**Table 6:** Association between Sudanese immigrants' socio-demographic data and quality of life

Items	N	Quality of life levels			X^2^	P
Age		Satisfactory 160 (41.6%)	Average 125 (32.5%)	Unsatisfactory 100 (25.9%)		
<25	110	47 (42.7%)	30 (27.2)	33 (30)	5.1	0.27
25-40	164	70 (42.6)	60 (36.5)	34 (20.7)		
>40	111	43 (38.7)	35 (31.5)	33 (29.7)		
Gender					20.9	0.0003
Male	225	115 (51.2)	64 (28.4)	46 (20.4)		
Female	160	45 (28.1)	61 (38.1)	54 (33.7)		
Educational level					54.5	0.00001
Primary	80	20 (25)	30 (37.5)	30 (37.5)		
Secondary	125	30 (24)	55 (44)	40 (32)		
University education	180	110 (61.1)	40 (22.2)	30 (16.6)		
How would you describe your current financial situation?						
Good	210	125 (59.5)	55 (26.1)	30 (14.2)	66.0	0.00001
Fair	95	20 (21.1)	40 (42.1)	35 (36.8)		
Poor	80	15 (18.7)	30 (37.5)	35(43.7)		
What is the main reason for coming to Egypt?						
Permanent residence	220	115 (52.3)	80 (36.4)	25 (11.3)	58.7	0.00001
A transitional period	165	45 (27.3)	45 (27.3)	75(45.4)		

## Discussion

The current study aimed to comprehensively explore the quality of life, health-seeking behavior, and perceived barriers to health services utilization among Sudanese immigrants in Egypt. The study findings reveal a nuanced perspective on the quality of life among Sudanese immigrants in Egypt, with notable variations across different domains. Approximately half of the respondents (47.5%) reported a satisfactory quality of life in the physical domain, underscoring a positive outlook on their physical well-being. However, only about one-third (38.4%) perceived their psychological well-being as satisfactory, highlighting potential challenges in this aspect. Similarly, around half of the participants reported a satisfactory quality of life in the social domain, indicating a more favorable assessment of their social experiences. When considering the overall quality of life, the results show a distribution of 41.6% reporting satisfactory, 32.4% average, and 26.0% unsatisfactory levels.

These findings align with previous studies, such as those by Maneze et al. and Brand et al., which reported that migrants generally scored lower in mental HrQoL compared to native populations but not in physical HrQoL [6,13]. However, differences emerged in the social domain quality of life between the current study and the cited literature. Notably, the variations may be attributed to factors such as language proficiency and the presence of relatives in Egypt, suggesting that social connections and linguistic familiarity play pivotal roles in shaping immigrants' perceptions of their social well-being.

The findings from the current study underscore the significance of various socio-demographic factors in shaping the quality of life among Sudanese immigrants in Egypt. Specifically, educational level, financial situation, gender, and the permanency of immigration were identified as significant factors influencing the participants' perceptions of their quality of life. This aligns with research conducted by [14], which noted that the quality of life among immigrants exhibits significant variation influenced by factors including education, economic conditions, and even skin color, particularly for those facing persecution.

Moreover, the present study's results find support in the work of Gagliardi et al. [15], reinforcing the association between socio-demographic factors and quality of life. Along the same lines, Akokuwebe et al. contributed to this body of evidence, highlighting the significant impact of gender, education, population group, income, employment, health insurance coverage, and healthcare services usage on quality of life [11]. However, a notable difference emerged regarding the influence of age because the previous study did not observe a significant association, unlike the current study's findings. The divergence in results regarding age may be attributed to the unique experiences of immigrants, including acculturation processes, financial situations, or discrimination, which interact with age in complex ways. The complex interplay of these factors underscores the importance of considering the multifaceted nature of immigrant experiences and the various elements that contribute to their overall quality of life.

The study's findings shed light on the prominent barriers to healthcare services sought by Sudanese migrants, with the highest percentages attributed to specific challenges. Limited awareness of available services emerged as the most prevalent barrier, followed by competing priorities like work, a lack of financial resources, long waiting hours in healthcare facilities, and stigma and discrimination. These findings align with research by Uansri et al., which highlighted financial constraints, including the affordability of healthcare and difficulty accessing funds through migrant health insurance, as major barriers to healthcare access among migrant workers [16]. The study further identified structural barriers, such as some health facilities opening only for emergency cases, and highlighted the profound impact of insufficient healthcare resources during the peak of positive cases. Cognitive barriers, including negative attitudes and diverse understandings of healthcare rights, were also noted. Language and communication barriers, along with a lack of information, played significant roles in impeding access to healthcare.

Similarly, the results parallel the work of Kuan et al. and Leijen et al., who identified three overarching barriers among migrants seeking healthcare services: language and information, sociocultural and economic factors, and policy and resource-related challenges [17,18]. These collective findings emphasize the multifaceted nature of barriers encountered by migrants in accessing healthcare, encompassing not only financial and structural aspects but also cognitive and sociocultural dimensions.

The suggestions provided by Sudanese immigrants to improve healthcare access, as revealed by the current study, underscore the importance of culturally competent healthcare providers, inclusive healthcare policies, flexible clinic hours, legal protections against discrimination, and clear announcements about available healthcare services. Notably, about two-thirds of the participants expressed a need for culturally competent healthcare providers, emphasizing the role of cultural sensitivity in enhancing patient satisfaction, adherence to treatment, and overall health outcomes, as supported by Ejike and Al-Rayes et al. [19,20]. This aligns with the broader understanding that culturally competent care is pivotal in addressing the unique needs of diverse populations.

The majority of immigrants advocating for inclusive healthcare policies reflect a desire for healthcare systems that are equitable and responsive to the diverse backgrounds and circumstances of immigrants. This resonates with the emphasis placed by Alarcon on the need for policies that address social determinants of health and reduce disparities, particularly among immigrant populations [21]. Additionally, the recognition of the importance of flexible clinic hours aligns with findings by Juárez et al., who suggested that flexible hours positively impact healthcare utilization, especially for populations facing barriers related to work schedules or transportation [22].

The comparatively lower percentage of Sudanese immigrants (18.2%) mentioning legal protections for non-discrimination highlights the need for increased awareness and advocacy in this regard. Nevertheless, the majority stressing the importance of clear announcements about available healthcare services echoes the findings of Ray et al. [23], emphasizing the role of communication in promoting awareness and accessibility.

The results of the study offer valuable insights into the health-seeking behaviors of the immigrant Sudanese population. Approximately one-fifth of those who visited health services engaged in self-medication, while 28.6% opted for alternative medicine. Additionally, 16.9% of respondents indicated a tendency to delay addressing health problems, consistent with the observations by [19] that immigrants often choose alternative options over visiting health services when feeling ill.

About half of the respondents in the study participated in periodic health screenings, and approximately a quarter reported following antenatal care for themselves or their family members. This aligns with the findings of Sarría-Santamera et al., who noted that immigrant populations had a lower tendency to use health services related to primary health facilities [24].

The overall summary of health-seeking behavior in the study indicates that less than half exhibited positive health-seeking behavior. This aligns with a study by Yendaw et al., reporting that around half of immigrants in North-Western Ghana exhibited positive health-seeking behavior [25]. However, the proportion of Sudanese migrants with positive health-seeking behavior was higher than other immigrants reported by Al-Rayes et al. and Chen et al. [20,26]. This difference may be attributed to the availability of facilities from the Egyptian state for Sudanese immigrants, coupled with the absence of official obstacles to using health services.

Recommendations

Implementing community-based health education programs plays a pivotal role in empowering immigrant Sudanese individuals in Egypt to make informed health choices and adopt healthy lifestyles. Disseminating information about available healthcare services is a crucial component of improving healthcare access for immigrant Sudanese individuals.

Limitations

While this study provides valuable insights into the quality of life, health-seeking behavior, and barriers to healthcare among immigrant Sudanese individuals in Egypt, it is essential to acknowledge its limitations. The cross-sectional design employed in this research captures a snapshot of the participants' experiences at a specific point in time, limiting their ability to establish causal relationships or discern temporal patterns. Longitudinal studies would offer a more comprehensive understanding of how these factors evolve over time among the immigrant population. Additionally, the reliance on self-reported data introduces the possibility of response bias, as participants may provide socially desirable responses or may recall information inaccurately.

## Conclusions

The overall quality of life among the immigrant Sudanese was diverse, with more than one-third reporting satisfactory levels, while a notable proportion indicated average or unsatisfactory levels. Gender, educational level, financial situation, and the main reason for immigration significantly influenced the reported quality of life. Positive health-seeking behavior was prevalent among more than half of the studied immigrants. However, there were various barriers to healthcare access, such as lack of financial resources, long waiting hours in healthcare facilities, competing priorities like work, limited awareness of available services, and discrimination.
